# Cancel cancer: The immunotherapeutic potential of CD200/CD200R blockade

**DOI:** 10.3389/fonc.2023.1088038

**Published:** 2023-01-23

**Authors:** Deborah Choe, Dongwon Choi

**Affiliations:** Department of Surgery, Norris Comprehensive Cancer Center, Keck School of Medicine of USC, University of Southern California, Los Angeles, CA, United States

**Keywords:** CD200, CD200R, immunotherapy, immune-checkpoint, tumor microenvironment, lymphatics

## Abstract

Immune checkpoint molecules function to inhibit and regulate immune response pathways to prevent hyperactive immune activity from damaging healthy tissues. In cancer patients, targeting these key molecules may serve as a valuable therapeutic mechanism to bolster immune function and restore the body’s natural defenses against tumors. CD200, an immune checkpoint molecule, is a surface glycoprotein that is widely but not ubiquitously expressed throughout the body. By interacting with its inhibitory receptor CD200R, CD200 suppresses immune cell activity within the tumor microenvironment, creating conditions that foster tumor growth. Targeting the CD200/CD200R pathway, either through the use of monoclonal antibodies or peptide inhibitors, has shown to be effective in boosting anti-tumor immune activity. This review will explore CD200 and the protein’s expression and role within the tumor microenvironment, blood endothelial cells, and lymph nodes. This paper will also discuss the advantages and challenges of current strategies used to target CD200 and briefly summarize relevant preclinical/clinical studies investigating the immunotherapeutic efficacy of CD200/CD200R blockade.

## 1 Introduction

The development and growth of a tumor are influenced by multiple factors in its surroundings, such as the activation of pro-metastatic pathways (1), the angiogenesis of tumor-nourishing blood vessels (2), and the presence of tumor-related inflammation (3), including the activity of tumor-specific T cells (4). The induction of these pro-tumorigenic events is modulated by specific cells known as tumor-associated myeloid cells (TAMCs) (5). Members of TAMCs include myeloid-derived suppressor cells (MDSCs), tumor-associated macrophages (TAMs) (6), and dendritic cells (DCs) (7). MDSCs, which are immature myeloid cells that are highly numbered in various tumors, are capable of downregulating anti-tumor immune activity *via* the release of different chemicals and factors (8). They include reactive oxygen species (ROS), nitric oxide (NO) synthase, arginase 1, and anti-inflammatory cytokines (8). Targeting the pathways that involve these select groups of cells may be valuable for exploring potential immunotherapy against cancers (7). One major player in the regulation of TAMCs and subsequently the growth of a tumor is CD200 (6), an immunomodulatory protein that binds to the CD200R receptor and downregulates immune cell activity (9).

Alternatively known as OX2, CD200 is a 41-47 kDa, highly conserved glycoprotein (10) that is widely expressed on a variety of cell types, including follicular DCs, B cells, T cells, thymocytes, endothelial cells, placental epithelial cells, kidney cells, and neuronal cells (11). By interacting with its inhibitory receptor CD200R, CD200 modulates immune cell activity by inhibiting proinflammatory cytokine secretion, enhancing anti-inflammatory cytokine secretion (12), and promoting both regulatory T cell (Treg) induction (13) and MDSC production (14). Likewise, the CD200/CD200R interaction suppresses the activities of both natural killer (NK) cells (15) and basophils (16). More specifically regarding NK cells, evidence from some cancer studies strongly suggests that CD200 activity directly suppresses the cytotoxic mechanisms of NK cells and their ability to produce interferon-gamma (IFN-γ) (15). Other cancer studies demonstrate the additional ability of the CD200/CD200R pathway to promote the apoptosis of NK cells (17).

The broad expression of CD200 is advantageous, as the body can regulate the protein’s levels accordingly and regionally manage immune activity and inflammation in specific areas (18). Although the effects of CD200/CD200R may decrease inflammation, it can also, unfortunately, suppress the body’s natural ability to combat tumors (19, 20),. This has been supported by previous studies which have discovered that high levels of CD200 were expressed on an array of cancer cells, including but not limited to various leukemias (21), malignant melanoma (22), and several neuroendocrine cancers (23). Furthermore, the gene encoding the CD200 protein has been acquired by various viruses, including the Kaposi’s sarcoma-associated herpesvirus (KSHV) (24) and rat cytomegalovirus (RCMV) (25), which allows these pathogens to favorably suppress their hosts’ antiviral immune mechanisms. Exploring the evolutionary advantage of viral CD200 orthologs, the expression and role of CD200 in different biological settings, and previous studies investigating CD200-averse immunotherapy may help us better understand CD200/CD200R’s candidacy as a safe and suitable target for cancer therapy.

## 2 CD200 and CD200R

Human CD200 is a transmembrane glycoprotein composed of 278 amino acids that is encoded by a gene on chromosome 3 (17) (26). CD200 belongs to the immunoglobulin superfamily (IgSF) of proteins (27). IgSF proteins distinctively possess at least one immunoglobulin (Ig)-like domain that is structurally composed of two antiparallel β-sheets held together by a disulfide bone (28). CD200 possesses two extracellular Ig-like domains, one transmembrane domain (18), and a 19-amino acid cytoplasmic region (29). This short intracellular “tail” does not contain any relevant motifs involved in signaling pathways (29).

Linked to the CD200 gene is the gene for CD200R, which is composed of 348 amino acids (26). CD200R is also an IgSF protein and possesses two extracellular Ig-like domains (18, 29). However, unlike CD200, CD200R has phosphorylatable tyrosine motifs present in its 67-amino acid cytoplasmic region, reflecting the receptor’s ability to participate in intracellular signaling (30). The last residue of the three tyrosine residues in this cytoplasmic region is part of an NPxY motif (31). In contrast with CD200’s broad expression, CD200R’s expression is limited to the surfaces of myeloid cells, including DCs, macrophages, and mast cells (32), as well as specific lymphoid cells (B cells and select T cells) (33). CD200R is especially highly expressed on the surfaces of neutrophils, macrophages (32), and basophils (16).

**Figure 1:**
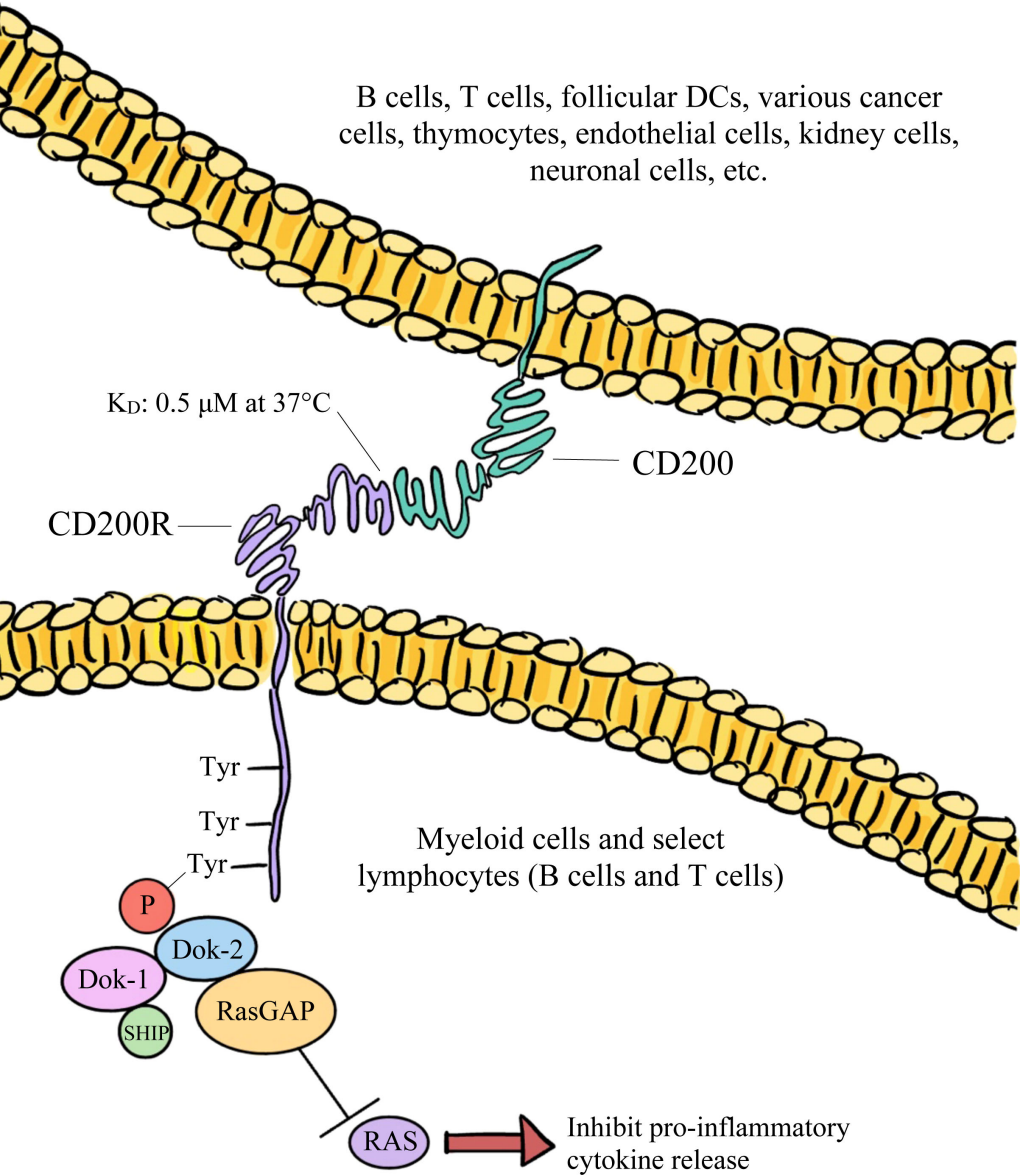
The broadly expressed CD200 glycoprotein predominantly interacts with its CD200R receptor and downregulates both the function and activation of immune cells *via* the inhibition of RAS signaling.

CD200 binds to CD200R with an equilibrium dissociation constant (K_D_) of approximately 0.5 μM at 37°C (32). This binding is facilitated by the proteins’ similar structures, resembling interactions that are involved in immunological synapses (18). Both being IgSF proteins, CD200 and CD200R interact through their shared NH_2_-terminal Ig-like domains (18, 27). Once CD200 binds, the third tyrosine residue associated with the NPxY motif within CD200R’s cytoplasmic tail becomes phosphorylated (31, 34). This results in the subsequent phosphorylation of tyrosine kinase 1 (DOK-1) and tyrosine kinase 2 (DOK-2), which are adaptor proteins (34). P hosphorylation of DOK-1 and DOK-2 leads to the binding of SHIP to DOK-1 and the recruitment of RasGAP, which negatively regulates the MAPK/ERK signaling pathway (34). A visual representation of the intracellular events that lead to RasGAP recruitment have been depicted in **Figure 1**. These events ultimately result in the inhibition of proinflammatory cytokine release and immune cell activation, such as the suppression of mast cell degranulation (34).

### 2.1 Soluble CD200

In addition to the form expressed on cellular surfaces, there are soluble forms of CD200 (known as sCD200) that are present in serum. A previous study investigating chronic lymphocytic leukemia (CLL) found that upregulated sCD200, and not the surface-expressed form of the immune checkpoint molecule, was correlated with worse tumor prognosis in patients with CLL (35). In addition, the engraftment of CLL cells from patients to immunocompromised mice was more successful in animals that had also received sCD200 plasma transfusions (35). In response to this study’s findings on sCD200, a separate group of researchers investigating glioblastoma multiforme (GBM) analyzed patients’ tumor sera and compared sCD200 levels among different types of brain tumors (14). They found that patients with GBM had significantly higher levels of sCD200 in sera compared to patients with ependymoma (14). Moreover, increased sCD200 levels were correlated with poor tumor prognoses in patients with GBM, ependymoma, and medulloblastoma. Additionally, investigators found that sCD200 was significantly associated with the expansion of MDSCs in patients with GBM (14). They suspected that sCD200 originating from tumors in the brain are able to travel to cervical lymph nodes *via* the cerebral spinal fluid and further suppress immune activity (14). The findings from both the CLL and GBM studies suggest that a soluble form of CD200 may further enhance immunosuppression and the development of select cancers.

### 2.2 CD200RL

Furthermore, CD200R is classified as a “paired receptor,” which means that the inhibiting receptor is associated with at least one other activating receptor (32, 36). It is thought that activating receptors arose from inhibiting receptors due to a gene duplication event (37). The activating sibling receptor for CD200R is known as CD200RL (for “CD200R-like”) (32). Humans only possess one form of CD200RL (known as CD200R1L), while mice can possess up to four different forms of the receptor (known as mCD200RLa, -b, -c, and -d) (32, 37). Despite sharing considerable sequence homology with CD200R (32), CD200R1L receptors do not bind to CD200 (38). Moreover, unlike in CD200R, the cytoplasmic regions present in CD200R1L are shorter and do not contain any signaling motifs (37). Rather, the transmembrane portions of activating receptors have charged amino acid residues that interact with adaptor proteins to deliver activating signals *via* YxxM or tyrosine-based activation motifs (37). Some studies suggest that CD200R1L may be linked to an increased risk for parasitic infections and skin conditions, including atopic dermatitis as well as psoriasis (37). Unfortunately, compared to CD200R, there are fewer studies exploring CD200RL and the receptor’s exact signaling mechanism and significance are not completely understood.

However, a study published in 2021 may represent a step forward in uncovering some of this information. Flow cytometry, Western blot, and qPCR analyses found that CD200R1L is expressed on human neutrophils in peripheral blood (37). Monoclonal antibody crosslinking of CD200R1L resulted in both significant production of ROS and interleukin-8 (IL-8), a chemotactic cytokine for neutrophils (37). These findings indicate that the activating receptors may serve a functional role on the surfaces of primary neutrophils in humans (37). Furthermore, sequence phylogeny suggests that the gene duplication event generating CD200RL likely occurred independently and multiple times among distantly related species and that the receptor has been conserved throughout evolution, indicating its biological importance in immunoregulation (37). Likewise, the faster mutation rate associated with the activating receptor relative to the inhibiting receptor led investigators to speculate that pathogenic pressure may have played a critical role in driving these mutations (37). This observation is consistent with CD200R1L-induced ROS production and the activating receptor’s anti-pathogenic role among neutrophils (37). Additionally, this data supports the “counterbalance theory” of paired receptors, which states that activating receptors may have evolved in hosts in response to pathogens that bind to inhibiting receptors (39). These activating receptors would thus serve a protective role by essentially “counterbalancing” the immunosuppressive signals triggered by pathogens capable of binding to inhibiting receptors (39).

### 2.3 Viral CD200 orthologs

Given CD200/CD200R’s ability to suppress immune activity, it is not surprising that the CD200-encoding gene has been acquired by a number of viruses, including the Shope (rabbit) fibroma virus (40), Yaba monkey tumor virus (41), human herpesvirus-6 (42), human herpesvirus-7 (16), and human herpesvirus-8 (43). From an evolutionary perspective, the acquisition of the gene for CD200 by many of these viruses is considered to have taken place independently among the various viral families according to their unique mechanisms of pathogenesis (24).

KSHV, also known as human herpesvirus-8, is a member of the *Gammaherpesviridae* subfamily (24) and the virus is necessary for the development of Kaposi sarcoma (KS) (44), a cancerous inflammatory cytokine disease (45) that is spread primarily *via* saliva (46). Unfortunately, in most cases, the cancer is not immediately detectable in the body as it develops after a period of latency following viral infection (47). KS commonly develops in HIV patients, suggesting that KS tumorigenesis may be enhanced under conditions of immunosuppression (48).

The KSHV ortholog of CD200, known as viral OX2 (vOX2), is a 55 kDa protein that is encoded by the virus’s K14 open reading frame (43). RNA analysis has shown that transcription of the viral gene is activated during KSHV’s replicative state, which is the lytic cycle (24). The vOX2 protein is found on the surfaces of cells infected with KSHV (43), which can range from B cells and DCs to epithelial cells and endothelial cells (48). The gene for vOX2 has approximately 40% sequence similarity with the human gene for CD200 (24). Although this level of sequence similarity is relatively low, vOX2 surprisingly shares key residues with CD200 in its binding site for CD200R (18). The viral ortholog also interacts with CD200R with a nearly identical binding affinity as that of CD200/CD200R (24). Through its binding to CD200R, vOX2 can effectively target host immune cells and inhibit anti-viral activity directed against the pathogen (49). For example, by binding to host CD200R, vOX2 can downregulate the activities of T cells (49), macrophages (24), neutrophils (50), and basophils (16). More specifically, vOX2 has been found to decrease the synthesis of the proinflammatory cytokines tumor necrosis factor-alpha (TNF-α) and IFN-γ from macrophages and T cells, respectively (49). The viral protein has also been shown to decrease the mobilization of CD107a (49), a marker for NK cell activity (49, 51).

Likewise, the CD200 ortholog for RCMV is known as the e127 protein and it binds to rat CD200R with a nearly identical affinity as the host ligand, despite only sharing 56% amino acid sequence similarity (25). Like vOX2, e127 has been shown to be expressed on the surfaces of infected cells (25). However, compared to viral orthologs, researchers suspect that the suppressive effects of e127 on host immune activity are more muted (25). Although both vOX2 and e127 successfully bind to host CD200R, the viral orthologs are not able to bind to respective CD200RL receptors (52). With the “counterbalance theory” in mind, researchers speculate that over time viruses capable of binding both inhibiting and activating host receptors eventually evolved to lose the ability to bind to activating receptors, which served as an evolutionary advantage for the pathogens (52).

Interestingly, one virus that is thought to have evolved further to ultimately lose its ability to interact with CD200R is the myxoma virus (MV). MV belongs to the *Chordopoxvirinae* subfamily and the virus’s natural hosts are rabbits (53). One study demonstrated that the lack of expression of the virus’s M141R protein in infected rabbits was associated with significant increases in the number of activated macrophages and NO production (53). These findings combined with analysis of sequence homology led investigators to strongly suspect that the M141R protein is an ortholog of CD200 (53). However, in another study, although M141 expression was also linked to downregulated immune activity and suppressed NO production, these effects could not be specifically attributed to mechanisms involving CD200R (54). Furthermore, compared to herpesvirus orthologs, it was found that the genetic and structural differences between poxvirus orthologs and their host CD200 proteins were more substantial (54). For example, poxvirus orthologs generally share lower sequence similarity with host CD200 compared to herpesvirus orthologs (54). Poxvirus orthologs also possess only one Ig-like domain rather than two (54). Overall, investigators concluded that M141 does not bind host CD200R and that the viral protein most likely exerts its immunosuppressive effects through a different signaling pathway (54). As a result, they also suggested that the CD200-similar sequences present in poxviruses most likely were acquired non-independently and thus evolved further among other poxvirus family members possessing such sequences (54).

### 2.4 CD200’s expression and role in the tumor microenvironment

**Figure 2:**
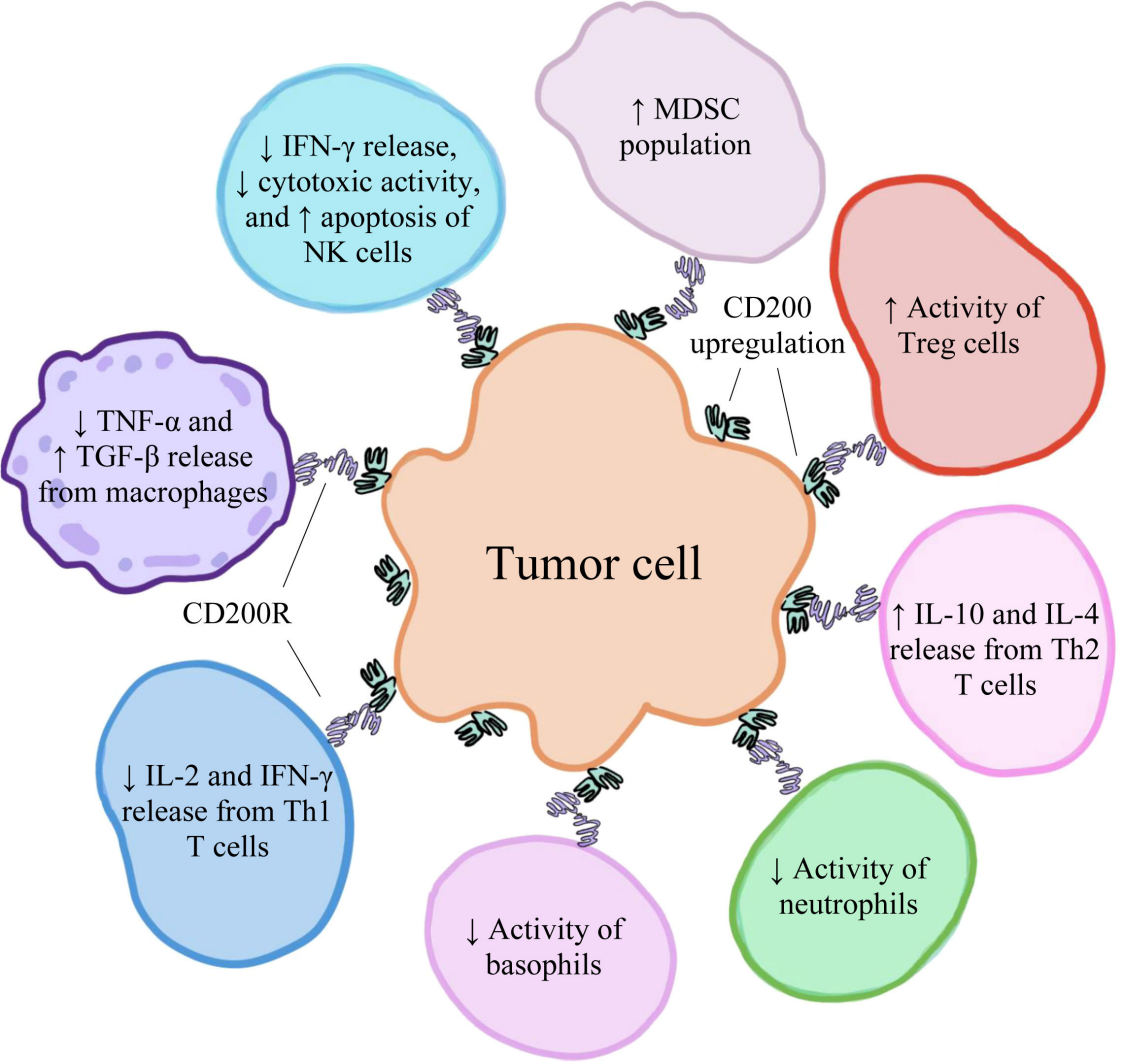
The upregulation of CD200 on various human cancer cells has an immunosuppressive effect on immune cells within the tumor microenvironment, creating conditions that favor tumor development, growth, and spread.

High levels of CD200 have been found to be expressed on various types of human cancer cells, including hairy cell leukemia (55), acute myeloid leukemia (56), malignant melanoma (22), CLL (21), multiple myeloma (21), testicular cancer (21), renal carcinoma (21), colon carcinoma (21), and GBM (57). Moreover, CD200 overexpression is correlated with poor prognosis among some of these cancers, suggesting that high levels of CD200 may facilitate tumorigenesis (21, 58–60). This observation can be explained by CD200’s primarily inhibitory role in the tumor microenvironment (TME) and the mechanisms through which the protein suppresses immune cell function and activation. In addition to tumor cells within the TME (21), CD200 is also predominantly found on T cells (30) and the endothelial cells lining tumor-nourishing blood vessels (61). In the TME, the soluble forms of CD200 are also present and can interact with CD200Rs (62), which are primarily found on TAMCs (including TAMs, TADCs, and MDSCs) (63). The binding of CD200 to CD200R results in increased induction of CD4^+^CD25^+^Foxp3^+^ Tregs, which act to negatively regulate immune responses (13). In addition to decreasing the activities of basophils (16) and NK cells (15), CD200/CD200R inhibits the release of TNF-α from activated macrophages expressing CD200R, suppressing the further activation of other macrophages (24). In macrophages, CD200/CD200R also upregulates the production of transforming growth factor-beta (TGF-β), an anti-inflammatory cytokine (64). The CD200/CD200R axis also decreases the release of interleukin-2 (IL-2) and IFN-γ, which are Th1-type proinflammatory cytokines, and increases the release of interleukin-10 (IL-10) and interleukin-4 (IL-4), which are Th2-type anti-inflammatory cytokines (12). Furthermore, this pathway increases the production of MDSCs (14). A visual representation of some of the various effects of CD200/CD200R on immune cell activity within the TME has been depicted in **Figure 2**. Therefore, the interactions between CD200 and CD200R in the TME negatively modulate levels of immune activity, ultimately fostering an environment that favors tumor development, growth, and spread (30).

One group of investigators examined the interaction between CD200 and MDSCs within the TME of pancreatic ductal adenocarcinoma (PDAC) (8). The researchers chose to study PDAC because the immunosuppression associated with the disease oftentimes makes the cancer resistant to immunotherapy (8). Immunofluorescence staining revealed that CD200 was highly expressed on tumor epithelial cells and on stromal cells within the PDAC TME (8). In addition, RNA from PDAC stromal cells was characterized as having significantly upregulated levels of CD200 compared to control RNA from normal pancreatic fibroblasts (8). Moreover, the researchers investigated CD200 expression on MDSCs in both cancer patients and healthy patients. Not only was there a higher number of MDSCs in cancer patients than in healthy patients but also a larger percentage of MDSCs expressing CD200R (8). The researchers wanted to further investigate the processes through which CD200 is able to regulate the population of MDSCs. Using single-cell RNA sequencing, they were able to isolate genes and downstream pathways, including those involved in interferon and cytokine signaling, that were highly upregulated in MDSCs expressing CD200R (8). Given these findings, the researchers suspected that by stimulating the expansion of the MDSC population, CD200 upregulation in the TME of PDAC potentially allows tumor cells to resist immunotherapy (8).

Another group of investigators examined the interactions between CD200 and NK cells within the TME of basal cell carcinoma (BCC) (17). They attributed the absence of NK cells in the BCC TME to sCD200, which is released from tumor cells *via* ectodomain shedding (17). The investigators demonstrated that CD200 downregulates NK cells’ anti-tumor activity by suppressing their activation, degranulation, and release of both cytokines and chemokines (17). More specifically, upon binding to CD200R on NK cells, sCD200 inhibits the MAPK/ERK pathway, suppressing tumor cell killing *via* the inhibition of IFN-γ release and promoting NK cell apoptosis *via* upregulated production of pro-apoptotic genes, including Fas, Fas ligand, and FADD (17). To demonstrate that CD200/CD200R negatively interferes with the MAPK/ERK pathway, the investigators cultured NK cells in the presence of CD200 (17). They observed a 4-fold decrease in both phosphorylated ERK 1 and ERK 2 in NK cells expressing CD200R but not in NK cells lacking CD200R (17). Furthermore, the researchers cultured mice neuronal cells expressing CD200R with human CD200 (17). They observed a decrease in phosphorylated ERK 1 and ERK 2 relative to overall ERK levels (17). To determine the mechanism through which CD200 promotes the apoptosis of NK cells, the investigators obtained NK cells from isolates of human peripheral blood mononuclear cells and introduced them to human CD200 (17). In the NK cells expressing CD200R, there was upregulated expression of Fas, Fas ligand, and FADD but not in the NK cells lacking CD200R (17). Overall, through these findings and those of additional experiments, this study effectively demonstrated that CD200 in the BCC TME suppresses NK cells’ cytotoxic activity and induces NK cell apoptosis, which foster immunosuppressed conditions favoring tumor development (17).

### 2.5 CD200’s expression and role in blood endothelial cells

The variable expression of CD200 in blood endothelial cells may further reveal the protein’s specific role in the vascular endothelium. Endothelial cells that line different blood vessels vary in their function and response to stress, including inflammation (65, 66). This is reflected by the cells’ variable expression of molecules like CD200, which has been found in blood vessels located in the lung (65), spleen (65), kidney (11), and fallopian tube (67). In a study involving rats, immunohistochemistry staining revealed that CD200 was strongly expressed on endothelial cells lining arterioles, the majority of veins (including venules), and some types of capillaries (65). However, expression was low in endothelial cells lining large arteries and the central veins located in the liver (65). The investigators speculated that varying levels of CD200 in different vessels may be attributed to environmental factors, including the force of blood flow and stimulatory factors released by cells from surrounding tissues (65, 68).

In the same study, the investigators also found that an anti-CD200 antibody disturbed the adhesion of T cells to human endothelial cells *in vitro*, suggesting that the interactions between CD200 and CD200R may facilitate the attachment of immune cells to the vessel endothelium (65). These findings suggest that variable factors like inflammatory stress may influence blood endothelial expression of CD200, which allows endothelial cells to successfully adhere to immune cells and deliver anti-inflammatory signals (65).

Furthermore, CD200 expression in blood endothelial cells may highlight how the protein’s presence can be strategically induced by certain metastasizing cancers. A study that investigated CD200 and CD200R expression in the TME of human squamous cell carcinoma (SCC) found that high levels of CD200 were expressed on endothelial cells lining the cancer’s vasculature (61). Additionally, it was found that the supernatant derived from the SCC tumor effectively induced the expression of CD200 on human dermal blood endothelial cells *in vitro* (61). The endothelial cells lining the vessels that surround and nourish tumors play a key role in enabling tumor metastasis, because the tumor cells must first pass through the endothelium before they are able to reach the circulatory system (69). Likewise, these endothelial cells are also important in the combating of tumors, because immune cells must first successfully pass through the endothelium of tumor vessels before they can access the tumor (61). Given this data, the investigators believe that the SCC tumor cells may favorably suppress immune activity in local blood endothelial cells by upregulating CD200 expression in these endothelial cells, facilitating the tumor’s entry into the circulatory system and, therefore, metastasis (61).

### 2.6 CD200’s expression and role in lymphatics

Lymphatic expression of CD200 also exhibits a degree of regional specificity. More specifically, CD200’s expression pattern among lymph node lymphatic endothelial cells (LECs) during inflammation may further highlight the protein’s specific immunomodulatory role within the lymphatic system (70). In addition to regulating the entrance and exit of immune cells from lymph nodes, lymph node LECs also facilitate the various interactions that transpire among different immune cells (70, 71). The organization of lymph node LECs further increase the lymph node’s efficiency in coordinating these interactions (70). For instance, within the lymph node subcapsular sinus (SCS), the space between the lymph node cortex and capsule, the LECs lining the sinus are further organized into ceiling and floor LECs (71). The SCS ceiling is the outward-facing wall of the sinus that is closest to the cortex, while the SCS floor is the inward-facing wall of the sinus that is closest to the parenchyma of the lymph node (71). These regional types of LECs uniquely respond to inflammation, which is further supported by their heterogeneous expression of molecules (70). In one murine study, inflammation that was similar to psoriasis was induced in mice ears by applying a cream containing imiquimod (70), a topical medicine used to treat genital warts and some skin cancers (72). Using single-cell RNA sequencing, investigators found that during skin inflammation, CD200 expression increased in SCS floor LECs of auricular lymph nodes (70). In normal skin, CD200 expression in LECs lining both the SCS ceiling and floor was variable (70). SCS resident macrophages and DCs, which are found in the floor endothelium (71), can be considered as the lymph node’s first layer of immune defense encountered by the incoming afferent lymph (73). Therefore, investigators speculated that CD200 upregulation in SCS floor LECs during inflammation may represent the body’s attempt to resolve inflammation at this protective frontline (70).

## 3 Targeting CD200/CD200R as immunotherapy

Considering CD200/CD200R’s ability to downregulate immune activity and suppress the body’s anti-tumor defenses, this cellular interaction has sparked researchers’ interest as being a key target for immunotherapy. Two currently explored mechanisms used to block the CD200/CD200R pathway include anti-CD200 monoclonal antibodies (51) and peptide CD200 inhibitors (57).

### 3.1 Anti-CD200 monoclonal antibody

Like CD200, other key immune checkpoint molecules that suppress immune cell function include cytotoxic T-lymphocyte-associated protein 4 (CTLA-4), programmed cell death protein 1 (PD-1), and T cell immunoglobulin-3 (Tim-3) (74). Monoclonal antibodies against these regulatory proteins have been developed, and their safety and efficacy as novel immunotherapy have been or are currently being investigated in clinical trials (74, 75). Some of these therapeutic monoclonal antibodies, including ipilimumab (anti-CTLA-4) and nivolumab (anti-PD-1), have already been approved by the FDA (76). Likewise, a monoclonal antibody that targets CD200 and prevents the ligand from binding to CD200R may effectively block the protein’s immunosuppressive signaling and restore the body’s protective defenses against tumor growth (51).

One study found that introduction of a human anti-CD200 monoclonal antibody was able to successfully enhance immune responses against acute myeloid leukemia (AML) (51). It was previously found that AML individuals with high levels of CD200 expression had reduced NK cell (15) and T cell activities (77), suggesting that CD200 overexpression had inhibited immune responses directed against AML cells (78). Based on these observations, the investigators developed a fully human anti-CD200 monoclonal antibody (designated “TTI-CD200”) to see if targeting CD200 could block the inhibitory signals transmitted to immune cells (51). When K562 human leukemia cells expressing high levels of CD200 (high-CD200) were exposed to regular NK cells *in vitro*, CD107a levels decreased in comparison to K562 cells expressing low levels of CD200 (low-CD200), confirming that the suppression of NK cell activity was CD200-related (51). However, TTI-CD200 introduction successfully restored NK cell activity to similar levels observed in low-CD200 K562 cells (51). Additionally, when high-CD200 K562 cells were cultured with TTI-CD200, the secretion of IFN-γ from NK cells increased (51). Investigators further took high-CD200 and low-CD200 AML blast cells from patients and cultured them with autologous lymphocytes (51). They exposed these cells to either TTI-CD200 or an isotope (control) (51). Compared to high-CD200 blast cells treated with the isotope, high-CD200 blast cells treated with the antibody exhibited increased levels of CD107a (51). On the other hand, low-CD200 blast cells did not show an increase in CD107a, regardless of receiving either the antibody or isotope treatment (51). Taken together, these findings demonstrate that the anti-CD200 monoclonal antibody effectively enhanced the previously diminished immune activity against myeloid leukemia cells *in vitro.*

In a Phase I clinical trial (NCT00648739) sponsored by Alexion Pharmaceuticals, a group of investigators tested the efficacy, safety, pharmacodynamics, and pharmacokinetics of samalizumab, a recombinant humanized anti-CD200 monoclonal antibody, in treating tumors associated with CLL and multiple myeloma (MM) (79). In the study, 26 patients (23 with CLL and 3 with MM) were each assigned one of seven different doses of samalizumab ranging from 50 to 600 mg/m^2^, which they planned to receive intravenously every 28 days (79). Only 21 of the 26 total patients ultimately received more than one treatment of samalizumab and were assessed at the end of the study (79). There was an observed dose-related reduction in the expression of CD200 on CLL cells following treatment (79). This decrease in expression was found to be longer-lasting in patients who received higher doses (300–500 mg/m^2^) of samalizumab compared to those who received lower doses (50–200 mg/m^2^) (79). Similarly, in both MM and CLL patients, there was a dose-related reduction in the presence of CD4^+^ T cells expressing CD200 but not in other T cell types (79). The tumor burden or the total amount of tumor present in the body was decreased in 14 CLL patients, which represented 64% of the total CLL patients who were assessed (79). However, in all MM patients, their disease had progressed over the course of the study (79). The maximum tolerated dose was not determined and the severity of any reported adverse events related to samalizumab treatment was only mild to moderate (79). Although the clinical trial was discontinued by Alexion Pharmaceuticals due to administrative reasons, the preliminary findings from the study demonstrate the relative safety of samalizumab and its potential to reduce the tumor load associated with CLL (79).

### 3.2 Peptide CD200 inhibitor

Compared to therapeutic monoclonal antibodies, using peptide inhibitors as immunotherapy has more advantages. Peptides are associated with lower toxicity (80), higher stability, and greater efficiency (81). They can better infiltrate cancerous tissues due to their smaller size (82) and they have high target precision, which means there is less risk for potential side effects (80). Additionally, peptides can be easily synthesized (80), altered (81), and are less expensive to produce compared to antibodies, making them a more favorable approach to cancer therapy (82).

Developing a peptide ligand that can effectively bind to CD200’s activating receptors may potentially block the inhibitory signals delivered by CD200/CD200R. It was previously found that tumor vascular endothelial cells in GBM had upregulated levels of CD200 expression (83). Through CD200 sequence analysis, investigators identified metalloprotease cleavage sites that, when cleaved, release peptides containing sequences compatible with CD200RL-binding (83). They were able to develop a peptide ligand (known as CD200AR-L) that could successfully bind to activating receptors, despite not fully understanding the exact mechanism of the synthetic ligand (83). However, one study may have identified the signaling pathway activated by CD200AR-L. Through *in vitro* studies, it was found that CD200AR-L stimulated immune activity by activating the DAP10 and DAP12 signaling pathways, which were transcriptionally upregulated in murine cells exposed to the peptide ligand (84). Additionally, investigators demonstrated *in vivo* that CD200AR-L downregulates the expression of CD200R1 in wild-type mice but not in mice lacking DAP10, which was found to be specifically involved in tumor growth regulation (84). Through stimulating these signaling pathways, the binding of CD200AR-L to activating receptors has been shown to increase cytokine secretion and boost anti-tumor defenses *via* the activation of antigen-presenting cells (APCs) (84). Overall, by interacting with activating receptors, CD200AR-L is able to block CD200’s inhibitory signaling by stimulating both DAP10/12 and downregulating expression of CD200R1 (84).

A study investigating high-grade glioma in mice found that administering CD200AR-L in addition to tumor-derived vaccines improved the animals’ immune response, which was originally diminished due to CD200 expression on cancer cells (57).

Both CD200R KO mice and wild-type mice received tumor-derived vaccines that contained either CD200AR-L or saline (control) (57). The two groups of mice that received vaccines containing CD200AR-L were found to have statistically significant increases in anti-tumor immune activity compared to those that received vaccines containing saline (57). Additionally, there was a significant increase in immune activity in wild-type mice that received the functional peptide ligand compared to those that received the ligand with its peptide sequence scrambled (57). The same group of investigators wanted to further assess CD200AR-L (20) and its effects in a study involving dogs diagnosed with spontaneous high-grade glioma (85). They found that, after tumor resection surgery, intradermal administration of canine CD200AR-L before tumor-lysate vaccination lengthened the dogs’ lifespans compared to the historical control group of dogs that only received the vaccine (median survival times = 12.9 months vs. 6.83 months) (85). This significant increase in post-surgery survival time for dogs receiving CD200AR-L was attributed to reduced CD200 expression on T cells and APCs and, therefore, their increased activities within the glioma TME (85).

Given the successful outcomes of both the murine and canine preclinical studies, the investigators developed humanized variations of CD200AR-L (designated “hCD200AR-L”) and tested their effects on human CD14^+^ cells (20). They ultimately chose to move forward with the hP1A8 peptide variant in a clinical trial (20). The hP1A8 form was able to effectively bind to immuno-activating receptors on CD14^+^ cells and upregulate immune activity by stimulating the activity of antigen-specific T cells, the maturation of DCs, and the production of proinflammatory cytokines (20). Furthermore, hP1A8 was associated with reduced CD200R expression, and it was also the analog of the most successful peptide tested in the prior murine study (20). In an ongoing Phase I clinical trial (NCT04642937) sponsored by OX2 Therapeutics, hP1A8’s therapeutic efficacy and appropriate dosage range are currently being studied (86). The peptide is being tested in conjunction with imiquimod and the GBM6-AD vaccine to treat GBM in 24 adult patients (86). The estimated date of study completion is November 2023 (86).

### 3.3 Risks and challenges of targeting CD200/CD200R

Despite efforts to investigate whether CD200-averse immunotherapy can be effectively applied to human patients, we must be mindful of existing limitations of the previously discussed CD200-targeting mechanisms. For instance, therapeutic peptides can have relatively short half-lives, low bioavailability, and the potential to be digested by enzymes present in the body (80). But, more importantly, there are other greater health risks that need to be acknowledged when considering CD200/CD200R blockade.

Because CD200 is expressed widely in the human body, targeting CD200/CD200R may have unintended consequences that can be especially harmful. For instance, targeting an immunomodulatory protein can impact the body’s ability to regulate inflammation during and following various infections and injuries. One study demonstrated that mice lacking CD200 expression had greater activity of lung macrophages and were thus more susceptible to prolonged influenza infection and eventual death (87). However, when the mice were treated with a CD200R agonist, they did not develop inflammatory lung disease (87). Another murine study demonstrated that blocking CD200R expression in animals was associated with higher risk of infection following ischemic stroke, greater movement of white blood cells into the brain, and increased mortality (88). This study demonstrated that blocking CD200 from binding to its inhibiting receptor interferes with the body’s ability to resolve inflammation stimulated by ischemic cerebral injury (88). Likewise, CD200 expression regulates levels of inflammation associated with various autoimmune diseases (19). The beneficial effects of CD200 were demonstrated in prior animal studies which found that the CD200/CD200R pathway lowered levels of inflammation and disease susceptibility in different autoimmune disease models, including those of arthritis and multiple sclerosis (89). Thus, it is possible that targeting the CD200/CD200R axis may lead to unintended hyperactive autoimmunity, which can significantly increase inflammation and damage otherwise healthy tissues (89). Furthermore, CD200 has shown to play a key role in the setting of transplants. CD200 expression is associated with a reduced likelihood of transplant rejection in mice (90). One study found that skin grafts from donor mice lacking either CD200 or CD200R1 had faster rates of graft rejection than those from control donor mice (91). Therefore, targeting CD200 may not be a viable option for patients receiving transplants.

## 4 Conclusion and future considerations

With all things considered, targeting CD200/CD200R has the potential to therapeutically bolster anti-tumor activity within the TME, surrounding blood vessels, and lymph nodes. However, further research and clinical studies are warranted to validate and reinforce CD200’s safety, efficacy, and accessibility as a target for immunotherapy. Not only are there limitations to current strategies used to target CD200, but there are overall health risks and potentially serious consequences that can arise from targeting an immunoregulatory protein that is broadly expressed. More specifically, blocking the CD200/CD200R pathway may result in hyperactive inflammation that could exacerbate the symptoms of cancer patients receiving immunotherapy. Furthermore, CD200-targeted immunotherapy may have varying success among patients depending on the type of cancer and their stage of disease progression, as demonstrated by the preliminary results of the samalizumab clinical trial (79). Additionally, it is worthwhile investigating the efficacy of CD200-targeted immunotherapy in conjunction with other forms of immunotherapy and cancer treatments. Simultaneously blocking other immune checkpoint molecules, such as CTLA-4, PD-1, and Tim-3, in addition to CD200 may synergistically boost anti-tumor activity. On the other hand, the effects of CD200-targeted immunotherapy may not be as successful if other immunosuppressive treatments, such as radiation or chemotherapy, are simultaneously administered. Nevertheless, given the current knowledge on CD200/CD200R, with further research, CD200-averse treatment shows promising potential to become the next FDA-approved immunotherapeutic drug.

## Author contributions

DChoe wrote the article and DChoi edited it. All authors contributed to the article and approved the submitted version.
